# Effect of Stochastic Loading on Tensile Damage and Fracture of Fiber-Reinforced Ceramic-Matrix Composites

**DOI:** 10.3390/ma13112469

**Published:** 2020-05-28

**Authors:** Longbiao Li

**Affiliations:** College of Civil Aviation, Nanjing University of Aeronautics and Astronautics, No.29 Yudao St., Nanjing 210016, China; llb451@nuaa.edu.cn

**Keywords:** ceramic-matrix composites (CMCs), Stochastic loading, tensile, matrix cracking, interface debonding, fiber failure

## Abstract

In this paper, the effect of stochastic loading on tensile damage and fracture of fiber-reinforced ceramic-matrix composites (CMCs) is investigated. A micromechanical constitutive model is developed considering multiple damage mechanisms under tensile loading. The relationship between stochastic stress, tangent modulus, interface debonding and fiber broken is established. The effects of the fiber volume, interface shear stress, interface debonding energy, saturation matrix crack spacing and fiber strength on tensile stress–strain curve, tangent modulus, interface debonding fraction and fiber broken fraction are analyzed. The experimental tensile damage and fracture of unidirectional and 2D SiC/SiC composites subjected to different stochastic loading stress are predicted. When fiber volume increases, the initial composite strain decreases, the initial tangent modulus increases, the transition stress for interface debonding decreases and the initial fiber broken fraction decreases. When fiber strength increases, the initial composite strain and fiber broken fraction decrease.

## 1. Introduction

Ceramic-matrix composites (CMCs) have the advantages of high-temperature resistance, corrosion resistance, low density, high specific strength and high specific modulus [1]. The fabrication methods of CMCs include the chemical vapor infiltration (CVI), polymer infiltration and pyrolysis process (PIP) and melt infiltration (MI). The mechanical performance of CMCs depends on the fabrication method. To ensure the reliability and safety of fiber-reinforced CMCs used in hot-section components of an aero engine, it is necessary to develop performance evaluation, damage evolution, strength and life prediction tools for airworthiness certification [2]. Since the applications of fiber-reinforced CMCs involve components with lives that are measured in tens of thousands of hours, the successful design and implementation of CMC components depend on the knowledge of the material behavior over periods of time comparable to the expected service life of the component [3].

Under tensile loading, multiple damage mechanisms of matrix cracking, interface debonding and fiber failure occur [4,5,6,7,8]. The tensile stress–strain curves can be divided into four stages, including:

(1) Stage I, linear-elastic region.
(2) Stage II, matrix cracking and interface debonding region. Matrix micro cracking occurs first and the fiber debonding from the matrix, leading to nonlinear behavior of CMCs.
(3) Stage III, saturation matrix cracking region. Matrix cracking approaches saturation with complete debonding of the fiber from the matrix.
(4) Stage IV, fiber failure region. The fiber gradually fractures with increasing applied stress.

The composite elastic modulus, proportional limit stress, ultimate tensile strength and the fracture strain can be obtained from the tensile stress–strain curve. For the SiC/SiC composite fabricated using the MI method, the fracture strength and strain of Hi-Nicalon S SiC/SiC composite is much higher than that of Tyranno SA3 SiC/SiC composite; however, the initial elastic modulus and proportional limit stress of Hi-Nicalon S SiC/SiC composite is lower than that of Tyranno SA3 SiC/SiC composite at the same fiber volume of 34.8% and the composite tensile strength increases with the fiber volume [9]. Marshall et al. [10] and Zok and Spearing [11] investigated the first matrix cracking and matrix multiple cracking evolution in fiber-reinforced CMCs using the fracture mechanics approach. Curtin [12] investigated multiple matrix cracking evolution of CMCs considering matrix internal flaws. Evans [13] developed an approach for design and life prediction issues for fiber-reinforced CMCs and established the relationship between macro mechanical behavior and constituent properties of CMCs. McNulty and Zok [14] investigated the low-cycle fatigue damage mechanism and established the damage models for predicting the low-cycle fatigue life of CMCs. Naslain et al. [15] investigated the monotonic and cyclic tensile behavior of Nicalon™ SiC/SiC minicomposite at room temperature. The relationship between the statistical parameters of both the fiber and the matrix and the fiber/matrix interfacial parameters and the effect of environment and the tensile curves has been established. Goto and Kagawa [16] investigated the tensile and fracture behavior of a bi-directional woven Nicalon™ SiC/SiC composite at room temperature. The tensile stress–strain curve shows nonlinear behavior above 70 MPa and the composite effective Young’s modulus at fracture was about 40% of the initial value. Guo and Kagawa [6] investigated the tensile fracture behavior and tensile mechanical properties of Nicalon™ and Hi-Nicalon™ SiC/BN/SiC composites at temperature between 298 and 1400 K in air atmosphere. The tensile strength dropped from 140 MPa at 800 K to 41 MPa at 1200 K. Meyer and Waas [17] investigated tensile response of notched SiC/SiC composite at elevated temperature using a novel digital image correlation technique. Li et al. [18,19,20] developed a micromechanical approach to predict the tensile behavior of unidirectional, cross-ply, 2D and 2.5D woven CMCs considering the damage mechanisms of matrix cracking, interface debonding and fiber failure. However, the effect of stochastic loading on tensile damage and fracture of fiber-reinforced CMCs has not been investigated.

The objective of this paper is to investigate the effect of stochastic loading on tensile damage and fracture of fiber-reinforced CMCs for the first time. A micromechanical constitutive model is developed considering multiple damage mechanisms under tensile loading. The relationship between stochastic stress, tangent modulus, interface debonding and fiber broken is established. The effects of fiber volume, interface shear stress, interface debonding energy, saturation matrix crack spacing and fiber strength on tensile stress–strain curve, tangent modulus, interface debonding fraction and fiber broken fraction are analyzed. The experimental tensile damage and fracture of unidirectional and 2D SiC/SiC composites subjected to different stochastic loading stress are predicted.

## 2. Theoretical Model

**Figure 1:**
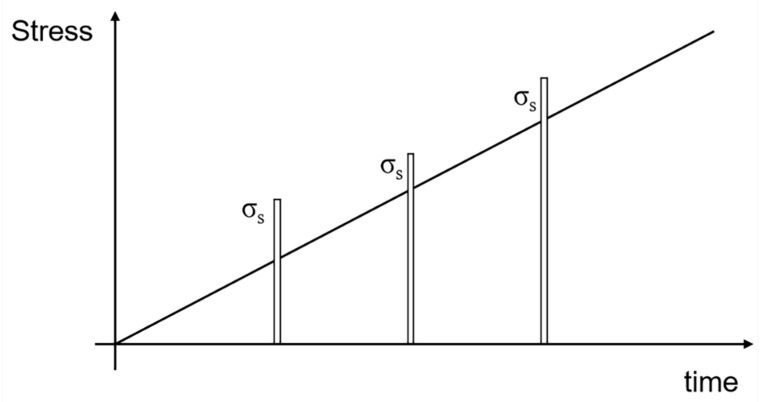
The diagram of stochastic loading under tensile loading.

**Figure 2:**
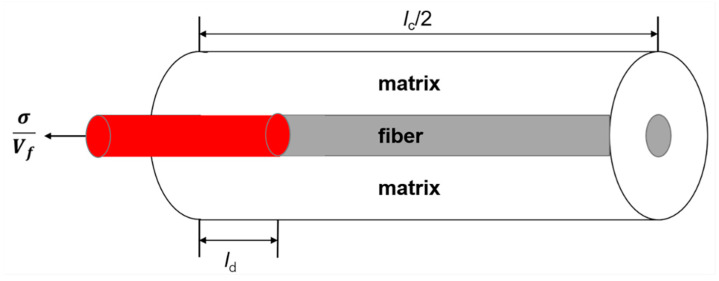
The unit cell of damaged composite.

When stochastic stress occurs under tensile loading, matrix cracking, interface debonding, and fiber failure occur. Multiple stochastic loading sequence is shown in Figure 1. The shear-lag model is used to analyze micro stress filed of damaged composite. A unit cell is extracted from the ceramic composite system, as shown in Figure 2. The fiber axial stress in different damage regions is given by Equation (1).

(1) $$\sigma_{f}\left(x\right)=\left\{ \begin{array}{l} \Phi -\frac{2\tau_{i}}{r_{f}}x,x\in \left[0,l_{d}\right]\\ \sigma_{\mathrm{fo}}+\left(\Phi -\sigma_{\mathrm{fo}}-2\frac{l_{d}}{r_{f}}\tau_{i}\right)\exp\left(-\rho \frac{x-l_{d}}{r_{f}}\right),x\in \left[l_{d},\frac{l_{c}}{2}\right] \end{array}\right.$$

where Φ is the fiber intact stress, *τ*_i_ is the interface shear stress, *r*_f_ is the fiber radius, *σ*_fo_ is the fiber axial stress in the interface bonding region, *l*_d_ is the interface debonding length, *l*_c_ is the matrix crack spacing and *ρ* is the shear-lag model parameter.

Using stochastic matrix cracking model, the relationship between stochastic stress and matrix crack spacing is given by Equation (2) [12].

(2) $$l_{c}=l_{s}{\left\{1-\exp\left[-{\left(\frac{\sigma_{m}}{\sigma_{R}}\right)}^{m}\right]\right\}}^{-1}$$

where *l*_s_ is the saturation matrix crack spacing, *σ*_m_ is the matrix stress, *σ*_R_ is matrix cracking characteristic stress and *m* is the matrix Weibull modulus.

The fracture mechanics approach is used to determine the fiber/matrix interface debonding length. The interface debonding length is determined by Equation (3).

(3) $$l_{d}=\frac{r_{f}}{2}\left(\frac{V_{m}E_{m}\sigma }{V_{f}E_{c}\tau_{i}}-\frac{1}{\rho }\right)-\sqrt{{\left(\frac{r_{f}}{2\rho }\right)}^{2}+\frac{r_{f}V_{m}E_{m}E_{f}}{E_{c}\tau_{i}^{2}}\zeta_{d}}$$

where *V*_f_ and *V*_m_ are the fiber and matrix volume fraction, respectively, *E*_m_ and *E*_c_ are the matrix and composite elastic modulus, respectively, and *ζ*_d_ is the interface debonding energy.

The Global Load Sharing (GLS) criterion is used to determine the intact fiber stress. The relationship between the fiber intact stress, fiber failure probability and fiber pullout length is given by Equation (4) [21].

(4) $$\frac{\sigma }{V_{f}}=\Phi \left(1-P\left(\Phi \right)\right)+\frac{2\tau_{i}}{r_{f}}\langle L\rangle P\left(\Phi \right)$$

where <*L*> is the average fiber pullout length, and *P*(Φ) is the fiber failure probability, which is obtained by Equation (5).

(5) $$P\left(\Phi \right)=1-\exp\left[-{\left(\frac{\Phi }{\sigma_{c}}\right)}^{m_{f}+1}\right]$$

where *σ*_c_ is the fiber characteristic strength, and *m*_f_ is the fiber Weibull modulus.

When matrix cracking and interface debonding occur, the composite strain is given by Equation (6).

(6) $$\varepsilon_{c}=\left\{ \begin{array}{c} \frac{\sigma }{V_{f}E_{f}}\eta -\frac{\tau_{i}}{E_{f}}\frac{l_{d}}{r_{f}}\eta +\frac{\sigma_{\mathrm{fo}}}{E_{f}}\left(1-\eta \right)-\frac{2}{\rho E_{f}}\left(\frac{V_{m}}{V_{f}}\frac{r_{f}}{l_{c}}\sigma_{\mathrm{mo}}-\eta \tau_{i}\right)\\ \;\;\;\times \left[\exp\left(-\frac{\rho }{2}\frac{l_{c}}{r_{f}}\left(1-\eta \right)\right)-1\right]-\left(\alpha_{c}-\alpha_{f}\right)\Delta T,\eta <1\\ \frac{\sigma }{V_{f}E_{f}}-\frac{1}{2}\frac{\tau_{i}}{E_{f}}\frac{l_{c}}{r_{f}}-\left(\alpha_{c}-\alpha_{f}\right)\Delta T,\eta =1 \end{array}\right.$$

When fiber failure occurs, the composite strain is given by Equation (7).

(7) $$\varepsilon_{c}=\left\{ \begin{array}{c} \frac{\Phi }{E_{f}}\eta -\frac{\tau_{i}}{E_{f}}\frac{l_{d}}{r_{f}}\eta +\frac{\sigma_{\mathrm{fo}}}{E_{f}}\left(1-\eta \right)-\frac{2}{\rho E_{f}}\left(\frac{r_{f}}{l_{c}}\left(\Phi -\sigma_{\mathrm{fo}}\right)-\eta \tau_{i}\right)\\ \;\;\;\times \left[\exp\left(-\frac{\rho }{2}\frac{l_{c}}{r_{f}}\left(1-\eta \right)\right)-1\right]-\left(\alpha_{c}-\alpha_{f}\right)\Delta T,\eta <1\\ \frac{\Phi }{E_{f}}-\frac{1}{2}\frac{\tau_{i}}{E_{f}}\frac{l_{c}}{r_{f}}-\left(\alpha_{c}-\alpha_{f}\right)\Delta T,\eta =1 \end{array}\right.$$

where *η* is the interface debonding fraction, as shown in Equation (8).

(8) $$\eta =2\frac{l_{d}}{l_{c}}$$

The tangent modulus is defined by Equation (9).

(9) $$E_{p}=\frac{d\sigma }{d\varepsilon }$$

## 3. Results and Discussion

Under stochastic loading, the damages of the matrix cracking, interface debonding and fiber failure occur. The micro stress filed of the damaged CMCs after stochastic loading is given by Equation (1). The fiber axial stress distribution is affected by the stochastic loading stress level, matrix cracking, interface debonding and fiber failure. The stochastic matrix cracking model is used to determine the matrix crack spacing at the applied stress level, as shown in Equation (2), and the fracture mechanics interface debonding criterion is used to determine the interface debonding length, and the GLS criterion is used to determine the load allocation between fracture and intact fibers. The micromechanical constitutive models for the conditions of the matrix cracking, interface debonding and fiber failure are given by Equations (6) and (7). Using the developed micromechanicam constitutive models and damage models, the effects of the fiber volume, interface shear stress, interface debonding energy, matrix crack spacing and fiber strength on tensile stress–strain curve, tangent modulus, interface debonding fraction and fiber broken fraction of SiC/SiC composite subjected to different stochastic loading are analyzed. Li et al. [4] investigated the tensile behavior of 2D woven SiC/SiC composite at room temperature. The composite was fabricated using the CVI method with the PyC interphase. The tensile experiments were conducted under displacement contrl of 0.3 mm/min. The material properties are given by: *V*_f_ = 0.2, *E*_f_ = 350 GPa, *E*_m_ = 300 GPa, *r*_f_ = 7.5 μm, *α*_f_ = 4.0 × 10^−6^/°C, *α*_m_ = 4.8 × 10^−6^/°C, ∆T = −1000 °C, *ζ*_d_ = 0.5 J/m^2^, *τ*_i_ = 25 MPa and *m*_f_ = 3.

### 3.1. Effect of Fiber Volume on Tensile Damage and Fracture of SiC/SiC Composite with Stochastic Loading

**Figure 3:**
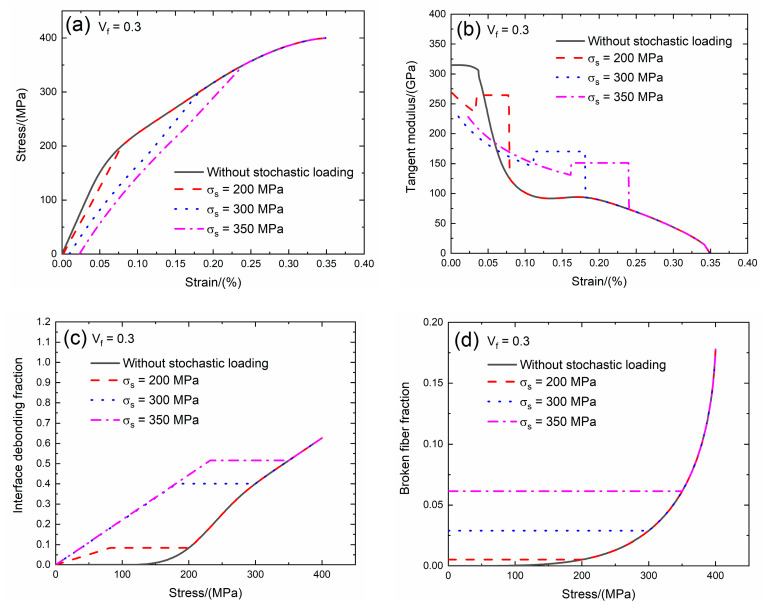
(**a**) The tensile stress–strain curves; (**b**) the tangent modulus versus strain curves; (**c**) the interface debonding fraction versus stress curves; and (**d**) the broken fibers fraction versus stress curves of SiC/SiC composite for conditions without stochastic loading and with stochastic loading of *σ*_s_ = 200, 300 and 350 MPa when *V*_f_ = 0.3.

**Figure 4:**
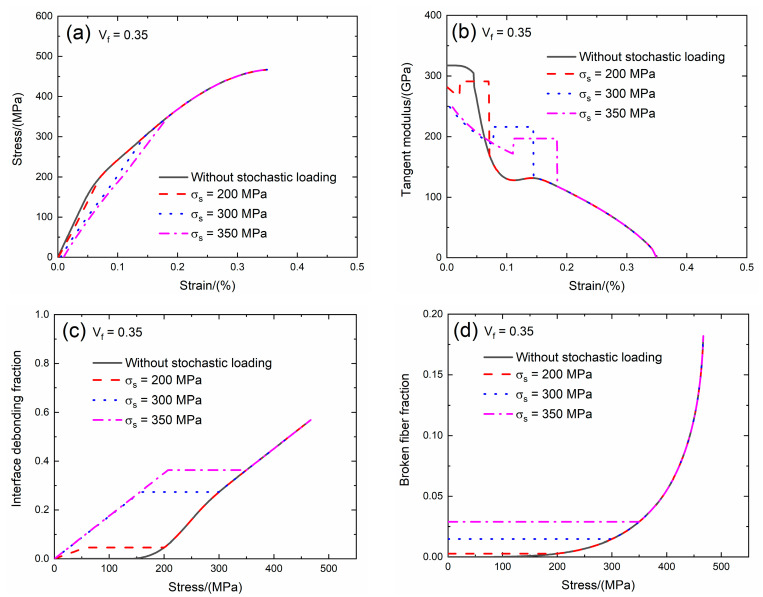
(**a**) The tensile stress–strain curves; (**b**) the tangent modulus versus strain curves; (**c**) the interface debonding fraction versus stress curves; and (**d**) the broken fibers fraction versus stress curves of SiC/SiC composite for conditions without stochastic loading and with stochastic loading of *σ*_s_ = 200, 300 and 350 MPa when *V*_f_ = 0.35.

The fiber volume affects the tensile behavior of CMCs. The fiber volume range of SiC/SiC composite is between *V*_f_ = 27.7 and 40% [9]. In the present analysis, the effect of the fiber volume (i.e., *V*_f_ = 0.3 and 0.35) on the tensile stress–strain curves, tangent modulus, interface debonding fraction and broken fiber fraction of SiC/SiC composite subjected to the stochastic loading of *σ*_s_ = 200, 300 and 350 MPa are shown in Figure 3; Figure 4 and Table 1. When the fiber volume increases, the stress carried by the fiber increases, the initial composite strain decreases, the initial tangent modulus increases, the transition stress for the interface debonding decreases and the initial fiber broken fraction decreases.

When *V*_f_ = 0.3 under *σ*_s_ = 200 MPa, the damages of matrix cracking and interface debonding occur at *σ*_s_ = 200 MPa, leading to the increase of the composite initial strain, decreasing of the tangent modulus and increase of the broken fiber fraction. The initial composite strain is *ε*_0_ = 0.00161% due to the matrix cracking and interface debonding at *σ*_s_ = 200 MPa; the initial tangent modulus is *E*_p_ = 268 GPa, the degradation rate of the tangent modulus is 15% compared with the original specimen and the fiber broken fraction is *P* = 0.005. With increasing stress to *σ*_tr_ = 79.2 MPa, the interface debonding fraction increases, the tangent modulus decreases to *E*_p_ = 235 GPa corresponding to *η* = 0.081. Upon increasing stress from *σ*_tr_ = 79.2 MPa to *σ* = 200 MPa, the tangent modulus remains constant of *E*_p_ = 264 GPa with *η* = 0.084.

Under *σ*_s_ = 300 MPa, the initial composite strain is *ε*_0_ = 0.00982% due to the damages of the matrix cracking and interface debonding at *σ*_s_ = 300 MPa; the initial tangent modulus is *E*_p_ = 229 GPa, the degradation rate of tangent modulus is 28% compared with the original specimen and the fiber broken fraction is *P* = 0.028. With increasing stress to *σ*_tr_ = 178.2 MPa, the interface debonding fraction increases, the tangent modulus decreases to *E*_p_ = 145 GPa corresponding to *η* = 0.392. Upon increasing stress from *σ*_tr_ = 178.2 MPa to *σ* = 300 MPa, the tangent modulus remains constant of *E*_p_ = 170.1 GPa with *η* = 0.4.

Under *σ*_s_ = 350 MPa, the initial composite strain is *ε*_0_ = 0.023% due to the damages of the matrix cracking and interface debonding at *σ*_s_ = 350 MPa; the initial tangent modulus is *E*_p_ = 228 GPa, the degradation rate of tangent modulus is 28% compared with the original specimen and the fiber broken fraction is *P* = 0.06. With increasing stress to *σ*_tr_ = 231 MPa, the interface debonding fraction increases, the tangent modulus decreases to *E*_p_ = 131 GPa corresponding to *η* = 0.51. Upon increasing stress from *σ*_tr_ = 131 MPa to *σ* = 350 MPa, the tangent modulus remains constant of *E*_p_ = 151 GPa with *η* = 0.51.

When *V*_f_ = 0.35 under *σ*_s_ = 200 MPa, the damages of matrix cracking and interface debonding occur at *σ*_s_ = 200 MPa, leading to the increase of the composite initial strain, decreasing of the tangent modulus and increase of the broken fiber fraction. The initial composite strain is *ε*_0_ = 0.0009%; the initial tangent modulus is *E*_p_ = 281 GPa, the degradation rate of tangent modulus is 12% compared with original specimen and the fiber broken fraction is *P* = 0.0027. With increasing stress to *σ*_tr_ = 55 MPa, the interface debonding fraction increases, the tangent modulus decreases to *E*_p_ = 265 GPa corresponding to *η* = 0.046. Upon increasing stress from *σ*_tr_ = 55 MPa to *σ* = 200 MPa, the tangent modulus remains constant of *E*_p_ = 291 GPa with *η* = 0.046.

Under *σ*_s_ = 300 MPa, the initial composite strain is *ε*_0_ = 0.0047% due to the damages of matrix cracking and interface debonding at *σ*_s_ = 300 MPa; the initial tangent modulus is *E*_p_ = 249 GPa, the degradation rate of tangent modulus is 22% compared with original specimen and the fiber broken fraction is *P* = 0.014. With increasing stress to *σ*_tr_ = 156.2 MPa, the interface debonding fraction increases and the tangent modulus decreases to *E*_p_ = 201 GPa corresponding to *η* = 0.27. When the stress increases from *σ*_tr_ = 156.2 MPa to *σ* = 250 MPa, the tangent modulus remains constant of *E*_p_ = 216 GPa with *η* = 0.27.

Under *σ*_s_ = 350 MPa, the initial composite strain is *ε*_0_ = 0.0095% due to the damages of the matrix cracking and interface debonding at *σ*_s_ = 350 MPa; the initial tangent modulus is *E*_p_ = 248 GPa, the degradation rate of tangent modulus is 22% compared with original specimen and the fiber broken fraction is *P* = 0.028. With increasing stress to *σ*_tr_ = 206.8 MPa, the interface debonding fraction increases and the tangent modulus decreases to *E*_p_ = 191.9 GPa corresponding to the interface debonding fraction *η* = 0.362. When the stress increases from *σ*_tr_ = 206.8 MPa to *σ* = 300 MPa, the tangent modulus remains constant of *E*_p_ = 196.7 GPa with *η* = 0.363.

### 3.2. Effect of Interface Shear Stress on Tensile Damage and Fracture of SiC/SiC Composite with Stochastic Loading

**Figure 5:**
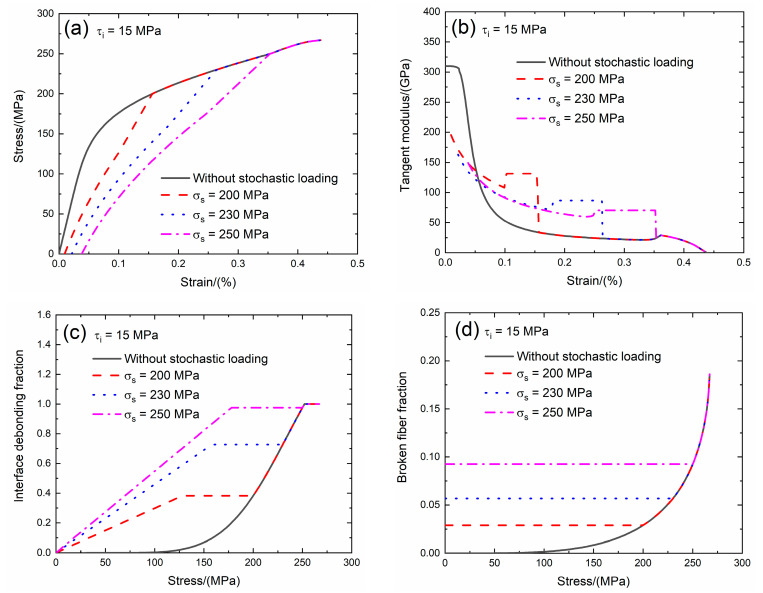
(**a**) The tensile stress–strain curves; (**b**) the tangent modulus versus strain curves; (**c**) the interface debonding fraction versus stress curves; and (**d**) the broken fibers fraction versus stress curves of SiC/SiC composite for conditions without stochastic loading and with stochastic loading of *σ*_s_ = 200, 230 and 250 MPa when *τ*_i_ = 15 MPa.

**Figure 6:**
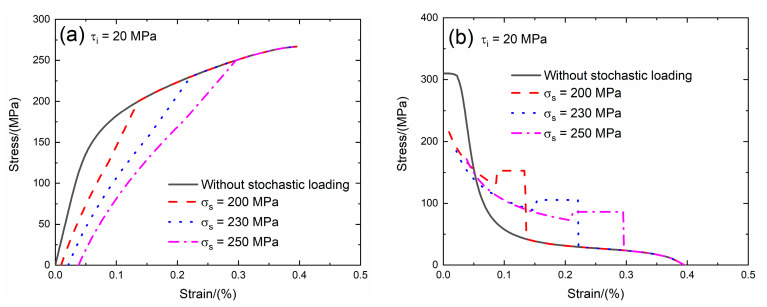

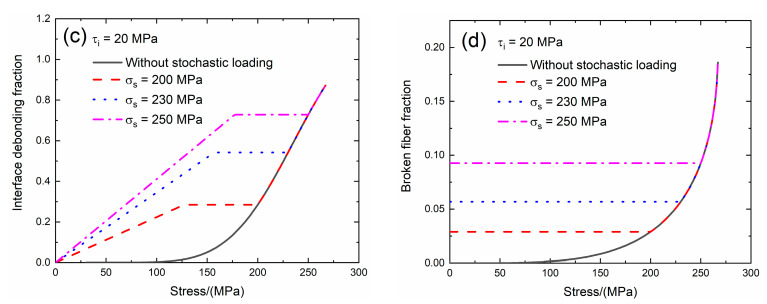
(**a**) The tensile stress–strain curves; (**b**) the tangent modulus versus strain curves; (**c**) the interface debonding fraction versus stress curves; and (**d**) the broken fibers fraction versus stress curves of SiC/SiC composite for conditions without stochastic loading and with stochastic loading of *σ*_s_ = 200, 230 and 250 MPa when *τ*_i_ = 20 MPa.

The interface shear stress transfers the load between the fiber and the matrix when the matrix cracking and interface debonding occur. For the weak interface bonding between the fiber and the matrix of SiC/SiC composite, the value of the interface shear stress is between *τ*_i_ = 10 and 30 MPa [22]. In the present analysis, the effect of the interface shear stress (i.e., *τ*_i_ = 15 and 20 MPa) on the tensile stress–strain curves, tangent modulus, interface debonding fraction and broken fiber fraction of SiC/SiC composite subjected to stochastic loading of *σ*_s_ = 200, 230 and 250 MPa are shown in Figure 5 and Figure 6 and Table 2. When the interface shear stress increases, the load transfer capacity between the fiber and the matrix increases, the initial composite strain remains the same, the initial tangent modulus increases, the transition stress for interface debonding remains the same and the initial fiber broken fraction remains the same.

When *τ*_i_ = 15 MPa under *σ*_s_ = 200 MPa, the damages of matrix cracking and interface debonding occur at *σ*_s_ = 200 MPa, leading to the increase of the composite initial strain, decreasing of the tangent modulus and increase of the broken fiber fraction. The initial composite strain is *ε*_0_ = 0.00943%; the initial tangent modulus is *E*_p_ = 195.6 GPa, the degradation rate of tangent modulus is 37% compared with original specimen and the fiber broken fraction is *P* = 0.028. With increasing stress to *σ*_tr_ = 125.4 MPa, the interface debonding fraction increases, the tangent modulus decreases to *E*_p_ = 108.1 GPa corresponding to *η* = 0.374. When the stress increases from *σ*_tr_ = 125.4 MPa to *σ* = 200 MPa, the tangent modulus remains constant of *E*_p_ = 131.3 GPa with *η* = 0.382.

Under *σ*_s_ = 230 MPa, the initial composite strain is *ε*_0_ = 0.021% due to the damages of matrix cracking and interface debonding at *σ*_s_ = 230 MPa; the initial tangent modulus is *E*_p_ = 163 GPa, the degradation rate of tangent modulus is 48% compared with original specimen and the fiber broken fraction is *P* = 0.056. With increasing stress to *σ*_tr_ = 154 MPa, the interface debonding fraction increases and the tangent modulus decreases to *E*_p_ = 71.5 GPa corresponding to *η* = 0.727. When the stress increases from *σ*_tr_ = 154 MPa to *σ* = 230 MPa, the tangent modulus remains constant of *E*_p_ = 86.8 GPa with *η* = 0.72.

Under *σ*_s_ = 250 MPa, the initial composite strain is *ε*_0_ = 0.038% due to the damages of the matrix cracking and interface debonding at *σ*_s_ = 250 MPa; the initial tangent modulus is *E*_p_ = 149.5 GPa, the degradation rate of the tangent modulus is 52% compared with original specimen and the fiber broken fraction is *P* = 0.092. With increasing stress to *σ*_tr_ = 173.8 MPa, the interface debonding fraction increases and the tangent modulus decreases to *E*_p_ = 60.7 GPa corresponding to *η* = 0.952. When the stress increases from *σ*_tr_ = 173.8 MPa to *σ* = 250 MPa, the tangent modulus remains constant of *E*_p_ = 70.4 GPa with *η* = 0.975.

When *τ*_i_ = 20 MPa under *σ*_s_ = 200 MPa, the damages of matrix cracking and interface debonding occur at *σ*_s_ = 200 MPa, leading to the increase of the composite initial strain, decreasing of the tangent modulus and increase of the broken fiber fraction. The initial composite strain is *ε*_0_ = 0.00943%; the initial tangent modulus is *E*_p_ = 215 GPa, the degradation rate of tangent modulus is 31% compared with original specimen and the fiber broken fraction is *P* = 0.028. With increasing stress to *σ*_tr_ = 123.2 MPa, the interface debonding fraction increases and the tangent modulus decreases to *E*_p_ = 130 GPa corresponding to *η* = 0.275. When the stress increases from *σ*_tr_ = 123.2 MPa to *σ* = 200 MPa, the tangent modulus remains constant of *E*_p_ = 152.9 GPa with *η* = 0.284.

Under *σ*_s_ = 230 MPa, the initial composite strain is *ε*_0_ = 0.021% due to the damages of the matrix cracking and interface debonding at *σ*_s_ = 230 MPa; the initial tangent modulus is *E*_p_ = 185 GPa, the degradation rate of the tangent modulus is 40% compared with original specimen and the fiber broken fraction is *P* = 0.056. With increasing stress to *σ*_tr_ = 154 MPa, the interface debonding fraction increases and the tangent modulus decreases to *E*_p_ = 88.6 GPa corresponding to *η* = 0.53. When the stress increases from *σ*_tr_ = 154 MPa to *σ* = 230 MPa, the tangent modulus remains constant of *E*_p_ = 105.5 GPa with *η* = 0.54.

Under *σ*_s_ = 250 MPa, the initial composite strain is *ε*_0_ = 0.038% due to the damages of the matrix cracking and interface debonding; the initial tangent modulus is *E*_p_ = 171.7 GPa, the degradation rate of tangent modulus is 45% compared with original specimen and the fiber broken fraction is *P* = 0.092. With increasing stress to *σ*_tr_ = 173.8 MPa, the interface debonding fraction increases and the tangent modulus decreases to *E*_p_ = 72.8 GPa corresponding to *η* = 0.714. When the stress increases from *σ*_tr_ = 173.8 MPa to *σ* = 250 MPa, the tangent modulus remains constant of *E*_p_ = 86.3 GPa with *η* = 0.73.

### 3.3. Effect of Interface Debonding Energy on Tensile Damage and Fracture of SiC/SiC Composite with Stochastic Loading

**Figure 7:**
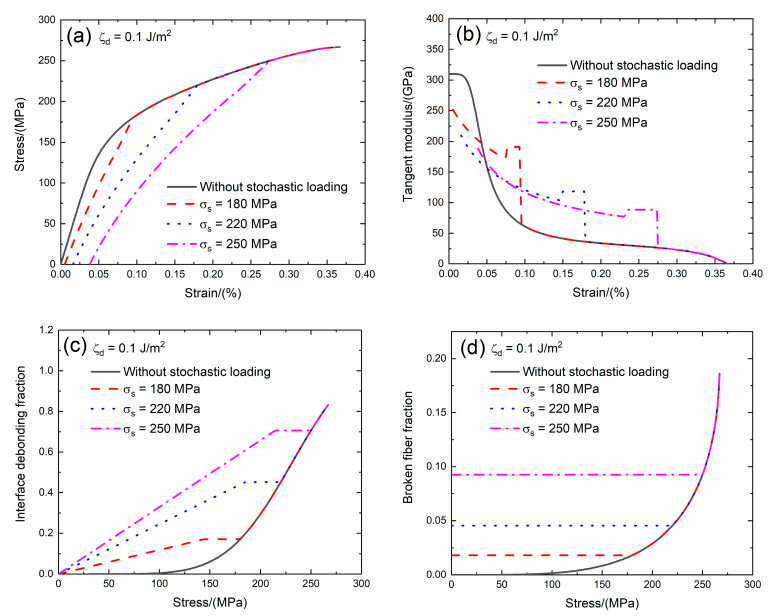
(**a**) The tensile stress–strain curves; (**b**) the tangent modulus versus strain curves; (**c**) the interface debonding fraction versus stress curves; and (**d**) the broken fibers fraction versus stress curves of SiC/SiC composite for conditions without stochastic loading and with stochastic loading of *σ*_s_ = 180, 220 and 250 MPa when ζ_d_ = 0.1 J/m^2^.

**Figure 8:**
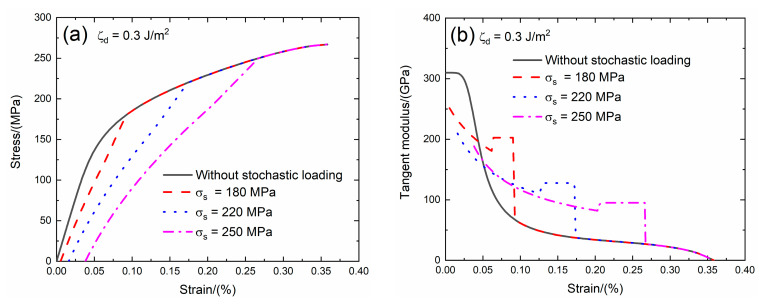

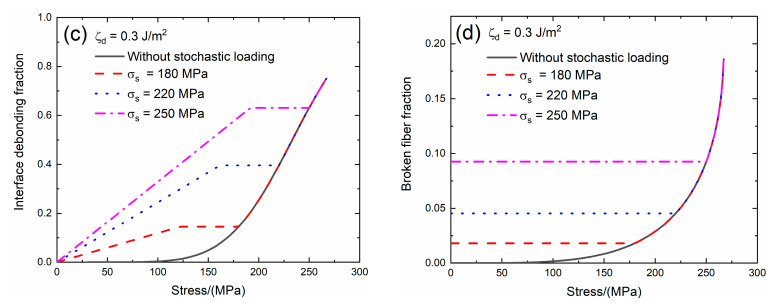
(**a**) The tensile stress–strain curves; (**b**) the tangent modulus versus strain curves; (**c**) the interface debonding fraction versus stress curves; and (**d**) the broken fibers fraction versus stress curves of SiC/SiC composite for conditions without stochastic loading and with stochastic loading of *σ*_s_ = 180, 220 and 250 MPa when ζ_d_ = 0.3 J/m^2^.

The interface debonding energy is a key interface property of CMCs. Domergue et al. [23] estimated the interface debonding energy of unidirectional SiC/CAS composite by analyzing the hysteresis loops and obtained the interface debonding energy is in the range of *ζ*_d_ = 0.1 – 0.8 J/m^2^. The effect of the interface debonding energy (i.e., *ζ*_d_ = 0.1 and 0.3 J/m^2^) on the tensile stress–strain curves, tangent modulus, interface debonding fraction and broken fiber fraction of SiC/SiC composite subjected to stochastic loading of *σ*_s_ = 180, 220 and 250 MPa are shown in Figure 7 and Figure 8 and Table 3. When the interface debonding energy increases, the initial composite strain, tangent modulus and broken fiber fraction remain the same, the transition stress for interface debonding decreases.

When *ζ*_d_ = 0.1 J/m^2^ under *σ*_s_ = 180 MPa, the damages of matrix cracking and interface debonding occur at *σ*_s_ = 180 MPa, leading to the increase of the composite initial strain, decreasing of the tangent modulus and increase of the broken fiber fraction. The initial composite strain is *ε*_0_ = 0.00537%; the initial tangent modulus is *E*_p_ = 251.5 GPa, the degradation rate of the tangent modulus is 19% compared with original specimen and the fiber broken fraction is *P* = 0.018. With increasing stress to *σ*_tr_ = 140.8 MPa, the interface debonding fraction increases and the tangent modulus decreases to *E*_p_ = 171.6 GPa corresponding to *η* = 0.167. When the stress increases from *σ*_tr_ = 140.8 MPa to *σ* = 180 MPa, the tangent modulus remains constant of *E*_p_ = 191 GPa with *η* = 0.171.

Under *σ*_s_ = 220 MPa, the initial composite strain is *ε*_0_ = 0.0161% due to the damages of the matrix cracking and interface debonding; the initial tangent modulus is *E*_p_ = 209 GPa, the degradation rate of the tangent modulus is 33% compared with original specimen and the fiber broken fraction is *P* = 0.045. With increasing stress to *σ*_tr_ = 182.6 MPa, the interface debonding fraction increases and the tangent modulus decreases to *E*_p_ = 103.6 GPa corresponding to *η* = 0.446. When the stress increases from *σ*_tr_ = 182.6 MPa to *σ* = 220 MPa, the tangent modulus remains constant of *E*_p_ = 118.4 GPa with *η* = 0.451.

Under *σ*_s_ = 250 MPa, the initial composite strain is *ε*_0_ = 0.038% due to the damages of the matrix cracking and interface debonding; the initial tangent modulus is *E*_p_ = 188.5 GPa, the degradation rate of the tangent modulus is 40% compared with the original specimen and the fiber broken fraction is *P* = 0.092. With increasing stress to *σ*_tr_ = 211.2 MPa, the interface debonding fraction increases and the tangent modulus decreases to *E*_p_ = 77 GPa corresponding to *η* = 0.694. When the stress increases from *σ*_tr_ = 211.2 MPa to *σ* = 250 MPa, the tangent modulus remains constant of *E*_p_ = 88.2 GPa with *η* = 0.705.

When *ζ*_d_ = 0.3 J/m^2^ under *σ*_s_ = 180 MPa, the damages of matrix cracking and interface debonding occur at *σ*_s_ = 180 MPa, leading to the increase of the composite initial strain, decreasing of the tangent modulus and increase of the broken fiber fraction. The initial composite strain is *ε*_0_ = 0.00537%; the initial tangent modulus is *E*_p_ = 251.5 GPa, the degradation rate of the tangent modulus is 19% compared with the original specimen and the fiber broken fraction is *P* = 0.018. With increasing stress to *σ*_tr_ = 118.8 MPa, the interface debonding fraction increases and the tangent modulus decreases to *E*_p_ = 180.6 GPa corresponding to *η* = 0.141. When the stress increases from *σ*_tr_ = 118.8 MPa to *σ* = 180 MPa, the tangent modulus remains constant of *E*_p_ = 202 GPa with *η* = 0.144.

Under *σ*_s_ = 220 MPa, the initial composite strain is *ε*_0_ = 0.0161%; the initial tangent modulus is *E*_p_ = 209 GPa, the degradation rate of tangent modulus is 33% compared with original specimen and the fiber broken fraction is *P* = 0.045. With increasing stress to *σ*_tr_ = 158.4 MPa, the interface debonding fraction increases and the tangent modulus decreases to *E*_p_ = 110.4 GPa corresponding to *η* = 0.387. When the stress increases from *σ*_tr_ = 158.4 MPa to *σ* = 220 MPa, the tangent modulus remains constant of *E*_p_ = 127.6 GPa with *η* = 0.396.

Under *σ*_s_ = 250 MPa, the initial composite strain is *ε*_0_ = 0.038% due to the damages of the matrix cracking and interface debonding; the initial tangent modulus is *E*_p_ = 188.5 GPa, the degradation rate of tangent modulus is 40% compared with the original specimen and the fiber broken fraction is *P* = 0.092. With increasing stress to *σ*_tr_ = 189.2 MPa, the interface debonding fraction increases and the tangent modulus decreases to *E*_p_ = 82 GPa corresponding to *η* = 0.622. When the stress increases from *σ*_tr_ = 189.2 MPa to *σ* = 250 MPa, the tangent modulus remains constant of *E*_p_ = 95.1 GPa with *η* = 0.63.

### 3.4. Effect of Saturation Matrix Crack Spacing on Tensile Damage and Fracture of SiC/SiC Composite with Stochastic Loading

**Figure 9:**
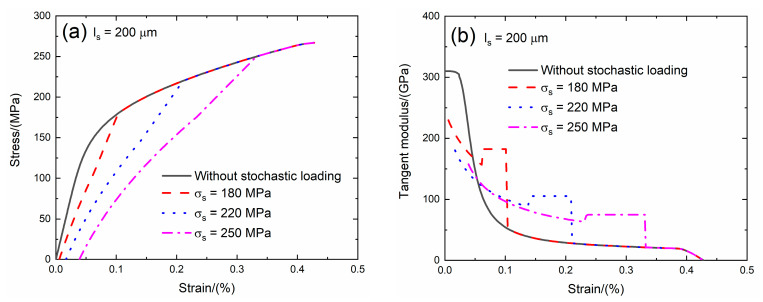

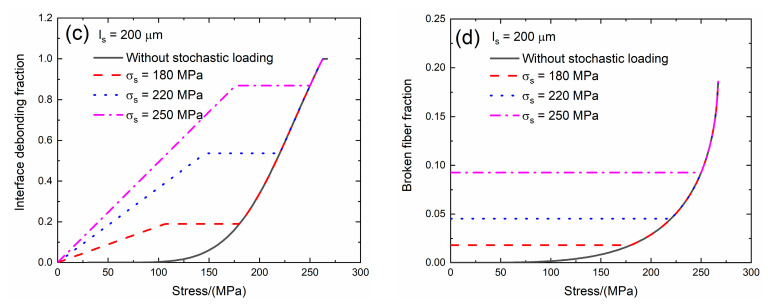
(**a**) The tensile stress–strain curves; (**b**) the tangent modulus versus strain curves; (**c**) the interface debonding fraction versus stress curves; and (**d**) the broken fibers fraction versus stress curves of SiC/SiC composite for conditions without stochastic loading and with stochastic loading of *σ*_s_ = 180, 220 and 250 MPa when *l*_s_ = 200 μm.

**Figure 10:**
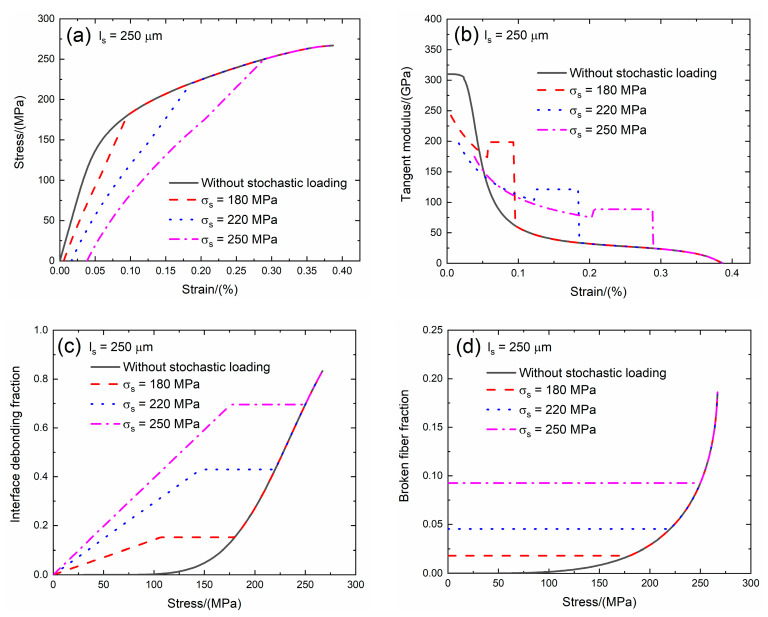
(**a**) The tensile stress–strain curves; (**b**) the tangent modulus versus strain curves; (**c**) the interface debonding fraction versus stress curves; and (**d**) the broken fibers fraction versus stress curves of SiC/SiC composite for conditions without stochastic loading and with stochastic loading of *σ*_s_ = 180, 220 and 250 MPa when *l*_s_ = 250 μm.

Li [24] investigated multiple matrix cracking of CMCs with different fiber preforms and found that the saturation matrix cracking spacing is in the range of *l*_s_ = 100 and 500 μm. In the present analysis, the effect of the saturation matrix crack spacing (i.e., *l*_s_ = 200 and 250 μm) on the tensile stress–strain curves, tangent modulus, interface debonding fraction and broken fiber fraction of SiC/SiC composite subjected to stochastic loading of *σ*_s_ = 180, 220 and 250 MPa are shown in Figure 9 and Figure 10 and Table 4. When saturation matrix crack spacing increases, the initial composite strain decreases, the initial tangent modulus increases, the transition stress for interface debonding and initial fiber broken fraction remain the same.

When *l*_s_ = 200 μm under *σ*_s_ = 180 MPa, the damages of matrix cracking and interface debonding occur at *σ*_s_ = 200 MPa, leading to the increase of the composite initial strain, decreasing of the tangent modulus and increase of the broken fiber fraction. The initial composite strain is *ε*_0_ = 0.00567% due to the damages of the matrix cracking and interface debonding; the initial tangent modulus is *E*_p_ = 229.8 GPa, the degradation rate of tangent modulus is 26% compared with the original specimen and the fiber broken fraction is *P* = 0.018. With increasing stress to *σ*_tr_ = 103.4 MPa, the interface debonding fraction increases and the tangent modulus decreases to *E*_p_ = 156.5 GPa corresponding to *η* = 0.184. When the stress increases from *σ*_tr_ = 103.4 MPa to *σ* = 180 MPa, the tangent modulus remains constant of *E*_p_ = 182.3 GPa with *η* = 0.189.

Under *σ*_s_ = 220 MPa, the initial composite strain is *ε*_0_ = 0.0168% due to the damages of the matrix cracking and the interface debonding; the initial tangent modulus is *E*_p_ = 180.3 GPa, the degradation rate of tangent modulus is 42% compared with original specimen and the fiber broken fraction is *P* = 0.045. With increasing stress to *σ*_tr_ = 143 MPa, the interface debonding fraction increases and the tangent modulus decreases to *E*_p_ = 88.1 GPa corresponding to *η* = 0.524. When the stress increases from *σ*_tr_ = 143 MPa to *σ* = 220 MPa, the tangent modulus remains constant of *E*_p_ = 105.2 GPa with *η* = 0.536.

Under *σ*_s_ = 250 MPa, the initial composite strain is *ε*_0_ = 0.038% due to the damages of the matrix cracking and the interface debonding; the initial tangent modulus is *E*_p_ = 157.7 GPa, the degradation rate of tangent modulus is 50% compared with original specimen and the fiber broken fraction is *P* = 0.092. With increasing stress to *σ*_tr_ = 173.8 MPa, the interface debonding fraction increases and the tangent modulus decreases to *E*_p_ = 64 GPa corresponding to *η* = 0.857. When the stress increases from *σ*_tr_ = 173.8 MPa to *σ* = 250 MPa, the tangent modulus remains constant of *E*_p_ = 75.3 GPa with *η* = 0.869.

When *l*_s_ = 250 μm under *σ*_s_ = 180 MPa, the damages of matrix cracking and interface debonding occur at *σ*_s_ = 180 MPa, leading to the increase of the composite initial strain, decreasing of the tangent modulus and increase of the broken fiber fraction. The initial composite strain is *ε*_0_ = 0.00549% due to the damages of the matrix cracking and the interface debonding; the initial tangent modulus is *E*_p_ = 242.3 GPa, the degradation rate of tangent modulus is 22% compared with original specimen and the fiber broken fraction is *P* = 0.018. With increasing stress to *σ*_tr_ = 103.4 MPa, the interface debonding fraction increases and the tangent modulus decreases to *E*_p_ = 173.7 GPa corresponding to *η* = 0.147. When the stress increases from *σ*_tr_ = 103.4.5 MPa to *σ* = 180 MPa, the tangent modulus remains constant of *E*_p_ = 198.7 GPa with *η* = 0.151.

Under *σ*_s_ = 220 MPa, the initial composite strain is *ε*_0_ = 0.0164% due to the damages of the matrix cracking and the interface debonding; the initial tangent modulus is *E*_p_ = 196.8 GPa, the degradation rate of tangent modulus is 37% compared with original specimen and the fiber broken fraction is *P* = 0.045. With increasing stress to *σ*_tr_ = 143 MPa, the interface debonding fraction increases and the tangent modulus decreases to *E*_p_ = 102.8 GPa corresponding to *η* = 0.42. When the stress increases from *σ*_tr_ = 143 MPa to *σ* = 220 MPa, the tangent modulus remains constant of *E*_p_ = 121.2 GPa with *η* = 0.43.

Under *σ*_s_ = 250 MPa, the initial composite strain is *ε*_0_ = 0.038% due to the damages of the matrix cracking and the interface debonding; the initial tangent modulus is *E*_p_ = 174.8 GPa, the degradation rate of tangent modulus is 44% compared with original specimen and the fiber broken fraction is *P* = 0.092. With increasing stress to *σ*_tr_ = 173.8 MPa, the interface debonding fraction increases and the tangent modulus decreases to *E*_p_ = 75 GPa corresponding to *η* = 0.685. When the stress increases from *σ*_tr_ = 173.8 MPa to *σ* = 250 MPa, the tangent modulus remains constant of *E*_p_ = 88.6 GPa with *η* = 0.695.

### 3.5. Effect of Fiber Strength on Tensile Damage and Fracture of SiC/SiC Composite with Stochastic Loading

**Figure 11:**
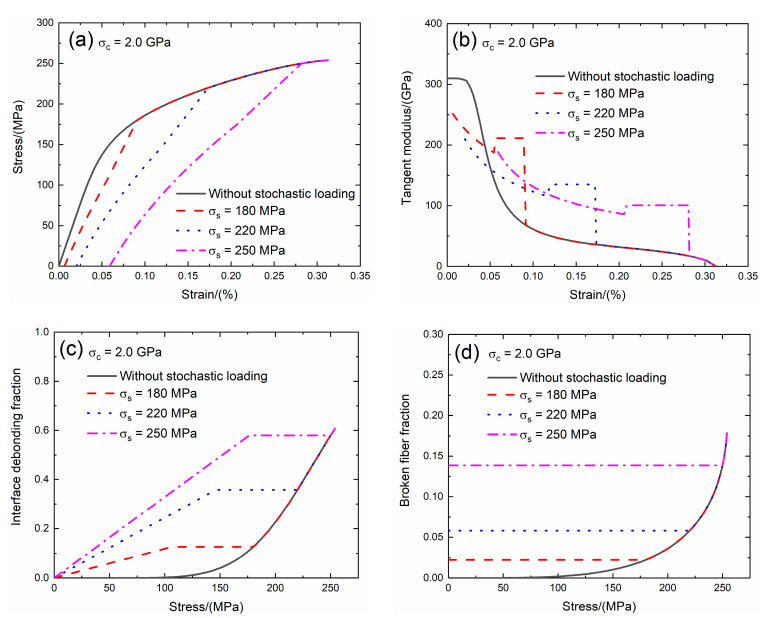
(**a**) The tensile stress–strain curves; (**b**) the tangent modulus versus strain curves; (**c**) the interface debonding fraction versus stress curves; and (**d**) the broken fibers fraction versus stress curves of SiC/SiC composite for conditions without stochastic loading and with stochastic loading of *σ*_s_ = 180, 220 and 250 MPa when *σ*_c_ = 2.0 GPa.

**Figure 12:**
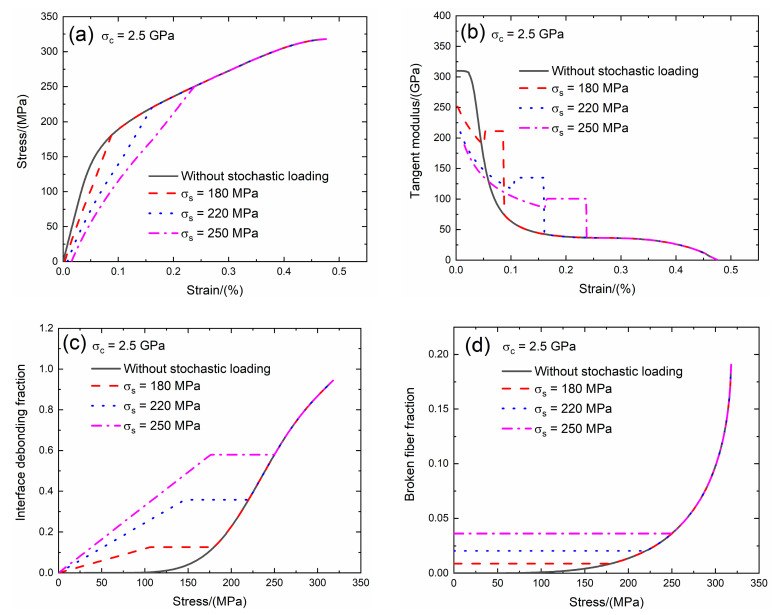
(**a**) The tensile stress–strain curves; (**b**) the tangent modulus versus strain curves; (**c**) the interface debonding fraction versus stress curves; and (**d**) the broken fibers fraction versus stress curves of SiC/SiC composite for conditions without stochastic loading and with stochastic loading of *σ*_s_ = 180, 220 and 250 MPa when *σ*_c_ = 2.5 GPa.

Guo et al. [25] investigated the SiC fiber strength and found that the SiC fiber strength is in the range between *σ*_c_ = 2.3 and 3.7 GPa. In the present analysis, the effect of the fiber strength (i.e., *σ*_c_ = 2.0 and 2.5 GPa) on the tensile stress–strain curves, tangent modulus, interface debonding fraction and broken fiber fraction of SiC/SiC composite subjected to stochastic loading of *σ*_s_ = 180, 220 and 250 MPa are shown in Figure 11 and Figure 12 and Table 5. When the fiber strength increases, the initial composite strain and fiber broken fraction decrease and the initial tangent composite modulus and transition stress for interface debonding remains the same.

When *σ*_c_ = 2.0 GPa under *σ*_s_ = 180 MPa, the damages of matrix cracking and interface debonding occur at *σ*_s_ = 180 MPa, leading to the increase of the composite initial strain, decreasing of the tangent modulus and increase of the broken fiber fraction. The composite initial strain is *ε*_0_ = 0.0065% due to the damages of the matrix cracking and the interface debonding; the initial tangent modulus is *E*_p_ = 251.5 GPa, the degradation rate of tangent modulus is 19% compared with original specimen and the fiber broken fraction is *P* = 0.022. With increasing stress to *σ*_tr_ = 103.4 MPa, the interface debonding fraction increases and the tangent modulus decreases to *E*_p_ = 187.4 GPa corresponding to *η* = 0.122. When the stress increases from *σ*_tr_ = 103.4 MPa to *σ* = 180 MPa, the tangent modulus remains constant of *E*_p_ = 211.3 GPa with *η* = 0.126.

Under *σ*_s_ = 220 MPa, the initial composite strain is *ε*_0_ = 0.02% due to the damages of the matrix cracking and the interface debonding; the initial tangent modulus is *E*_p_ = 209.6 GPa, the degradation rate of tangent modulus is 33% compared with original specimen and the fiber broken fraction is *P* = 0.058. With increasing stress to *σ*_tr_ = 143 MPa, the interface debonding fraction increases and the tangent modulus decreases to *E*_p_ = 115.7 GPa corresponding to *η* = 0.349. When the stress increases from *σ*_tr_ = 143 MPa to *σ* = 220 MPa, the tangent modulus remains constant of *E*_p_ = 134.9 GPa with *η* = 0.357.

Under *σ*_s_ = 250 MPa, the initial composite strain is *ε*_0_ = 0.059%; the initial tangent modulus is *E*_p_ = 188.5 GPa, the degradation rate of tangent modulus is 40% compared with original specimen and the fiber broken fraction is *P* = 0.138. With increasing stress to *σ*_tr_ = 173.8 MPa, the interface debonding fraction increases and the tangent modulus decreases to *E*_p_ = 86 GPa corresponding to *η* = 0.571. When the stress increases from *σ*_tr_ = 173.8 MPa to *σ* = 250 MPa, the tangent modulus remains constant of *E*_p_ = 100.6 GPa with *η* = 0.579.

When *σ*_c_ = 2.5 GPa under *σ*_s_ = 180 MPa, the damages of matrix cracking and interface debonding occur at *σ*_s_ = 180 MPa, leading to the increase of the composite initial strain, decreasing of the tangent modulus and increase of the broken fiber fraction. The initial composite strain is *ε*_0_ = 0.0028% due to the damages of the matrix cracking and the interface debonding; the initial tangent modulus is *E*_p_ = 251.5 GPa, the degradation rate of tangent modulus is 19% compared with original specimen and the fiber broken fraction is *P* = 0.0087. With increasing stress to *σ*_tr_ = 103.4 MPa, the interface debonding fraction increases and the tangent modulus decreases to *E*_p_ = 187.4 GPa corresponding to *η* = 0.122. When the stress increases from *σ*_tr_ = 103.4 MPa to *σ* = 180 MPa, the tangent modulus remains constant of *E*_p_ = 211.3 GPa with *η* = 0.126.

Under *σ*_s_ = 220 MPa, the initial composite strain is *ε*_0_ = 0.007% due to the damages of the matrix cracking and the interface debonding; the initial tangent modulus is *E*_p_ = 209.6 GPa, the degradation rate of tangent modulus is 33% compared with original specimen and the fiber broken fraction is *P* = 0.02. With increasing stress to *σ*_tr_ = 143 MPa, the interface debonding fraction increases and the tangent modulus decreases to *E*_p_ = 115.7 GPa corresponding to *η* = 0.344. When the stress increases from *σ*_tr_ = 140.8 MPa to *σ* = 220 MPa, the tangent modulus remains constant of *E*_p_ = 134.9 GPa with *η* = 0.357.

Under *σ*_s_ = 250 MPa, the initial composite strain is *ε*_0_ = 0.015%; the initial tangent modulus is *E*_p_ = 188.5 GPa, the degradation rate of the tangent modulus is 40% compared with original specimen and the fiber broken fraction is *P* = 0.036. With increasing stress to *σ*_tr_ = 173.8 MPa, the interface debonding fraction increases and the tangent modulus decreases to *E*_p_ = 86 GPa corresponding to *η* = 0.571. When the stress increases from *σ*_tr_ = 173.8 MPa to *σ* = 250 MPa, the tangent modulus remains constant of *E*_p_ = 100.6 GPa with *η* = 0.579.

## 4. Experimental Comparisons

Li et al. [4], Liu [5], Guo and Kagawa [6] and Morscher [7] investigated tensile behavior of unidirectional and 2D SiC/SiC composites at room temperature. In this section, using the developed damage models and micromechanical constitutive models for the conditions of matrix cracking, interface debonding and fiber failure, the experimental tensile stress–strain curves are predicted. The comparisons between tensile stress–strain curves with and without stochastic loading are analyzed. The relationships between the stochastic loading stress levels, tangent modulus, interface debonding fraction and fiber broken fraction are established.

### 4.1. 2D SiC/SiC under Stochastic Loading of 140, 180, 200 and 240 MPa

**Figure 13:**
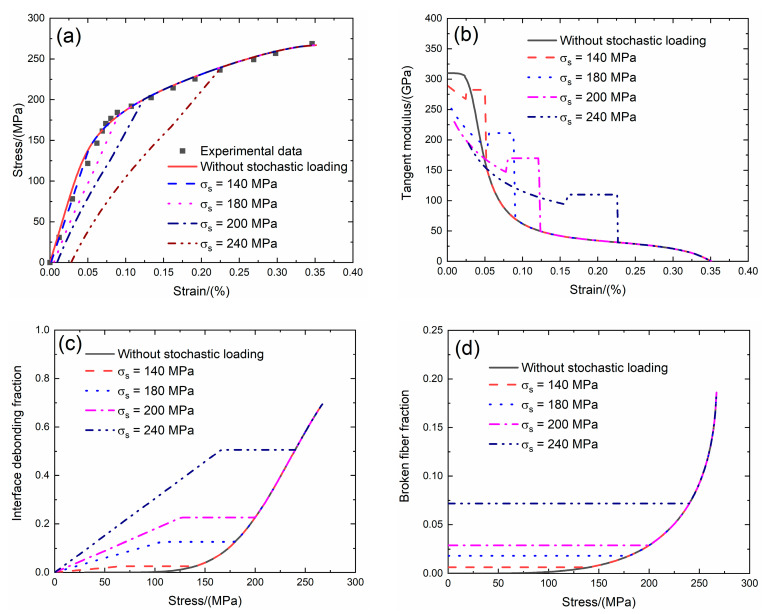
(**a**) The tensile stress–strain curves; (**b**) the tangent modulus versus strain curves; (**c**) the interface debonding fraction versus stress curves; and (**d**) the broken fibers fraction versus stress curves of 2D SiC/SiC composite for conditions without stochastic loading and with stochastic loading of *σ*_s_ = 140, 180, 200 and 240 MPa.

Li et al. [4] investigated the tensile behavior of 2D SiC/SiC composite at room temperature. The composite was fabricated using chemical vapor infiltration (CVI) method. The tensile test was performed under displacement control with the speed of 0.3 mm/min. The experimental tensile stress–strain curves, tangent modulus versus strain curves, interface debonding fraction and broken fiber fraction versus stress curves of 2D SiC/SiC composite without stochastic loading and with stochastic loading at *σ*_s_ = 140, 180, 200 and 240 MPa at room temperature are shown in Figure 13 and Table 6. When stochastic loading stress increases, the initial composite strain increases, the initial tangent modulus decreases, the transition stress for interface debonding increases and the initial fiber broken fraction increases.

Under *σ*_s_ = 140 MPa, the initial strain is *ε*_0_ = 0.001%; the initial tangent modulus is *E*_p_ = 288 GPa, the degradation rate of tangent modulus is 7% compared with original specimen and the fiber broken fraction is *P* = 0.006. With increasing stress to *σ*_tr_ = 63.8 MPa, the tangent modulus decreases to *E*_p_ = 267.4 GPa corresponding to *η* = 0.024. When the stress increases from *σ*_tr_ = 63.8 MPa to *σ* = 140 MPa, the tangent modulus remains constant of *E*_p_ = 282.3 GPa with *η* = 0.025.

Under *σ*_s_ = 180 MPa, the initial strain is *ε*_0_ = 0.005%; the initial tangent modulus is *E*_p_ = 251 GPa, the degradation rate of tangent modulus is 19% compared with original specimen and the fiber broken fraction is *P* = 0.018. With increasing stress to *σ*_tr_ = 103.4 MPa, the tangent modulus decreases to *E*_p_ = 187.4 GPa corresponding to *η* = 0.122. When the stress increases from *σ*_tr_ = 103.4 MPa to *σ* = 180 MPa, the tangent modulus remains constant of *E*_p_ = 211.3 GPa with *η* = 0.126.

Under *σ*_s_ = 200 MPa, the initial strain is *ε*_0_ = 0.009%; the initial tangent modulus is *E*_p_ = 229.5 GPa, the degradation rate of tangent modulus is 26% compared with original specimen and the fiber broken fraction is *P* = 0.028. With increasing stress to *σ*_tr_ = 123.2 MPa, the tangent modulus decreases to *E*_p_ = 147.1 GPa corresponding to *η* = 0.22. When the stress increases from *σ*_tr_ = 123.2 MPa to *σ* = 200 MPa, the tangent modulus remains constant of *E*_p_ = 169.7 GPa with *η* = 0.226.

Under *σ*_s_ = 240 MPa, the initial strain is *ε*_0_ = 0.028%; the initial tangent modulus is *E*_p_ = 194.2 GPa, the degradation rate of tangent modulus is 38% compared with original specimen and the fiber broken fraction is *P* = 0.078. With increasing stress to *σ*_tr_ = 162.8 MPa, the tangent modulus decreases to *E*_p_ = 94 GPa corresponding to *η* = 0.49. When the stress increases from *σ*_tr_ = 162.8 MPa to *σ* = 240 MPa, the tangent modulus remains constant of *E*_p_ = 109.8 GPa with *η* = 0.5.

### 4.2. UD and 2D SiC/SiC under Stochastic Loading

Liu [5] investigated the tensile behavior of unidirectional and 2D SiC/SiC composites at room temperature. The tensile test was performed under displacement control with the loading rate of 0.2 mm/min.

**Figure 14:**
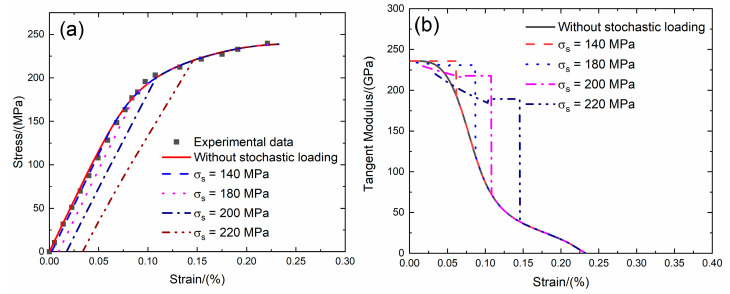

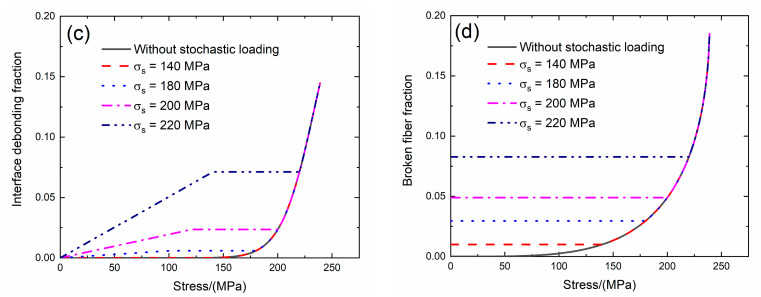
(**a**) The tensile stress–strain curves; (**b**) the tangent modulus versus strain curves; (**c**) the interface debonding fraction versus stress curves; and (**d**) the broken fibers fraction versus stress curves of UD SiC/SiC composite for conditions without stochastic loading and with stochastic loading of *σ*_s_ = 140, 180, 200 and 220 MPa.

For unidirectional SiC/SiC composite, the tensile stress–strain curves, tangent modulus versus strain curves, interface debonding fraction and broken fiber fraction versus stress curves without stochastic loading and with stochastic loading at *σ*_s_ = 140, 180, 200 and 220 MPa at room temperature are shown in Figure 14 and Table 7.

Under *σ*_s_ = 140 MPa, the initial strain is *ε*_0_ = 0.002%; the initial tangent modulus is *E*_p_ = 235.9 GPa, the degradation rate of tangent modulus is 1% compared with original specimen and the fiber broken fraction is *P* = 0.01. With increasing stress to *σ*_tr_ = 58.8 MPa, the tangent modulus decreases to *E*_p_ = 235.7 GPa corresponding to *η* = 0.0001. When the stress increases from *σ*_tr_ = 58.8 MPa to *σ* = 140 MPa, the tangent modulus remains constant of *E*_p_ = 235.8 GPa with *η* = 0.0001.

Under *σ*_s_ = 180 MPa, the initial strain is *ε*_0_ = 0.009%; the initial tangent modulus is *E*_p_ = 234 GPa, the degradation rate of tangent modulus is 1.1% compared with original specimen and the fiber broken fraction is *P* = 0.03. With increasing stress to *σ*_tr_ = 98.4 MPa, the tangent modulus decreases to *E*_p_ = 230.1 GPa corresponding to *η* = 0.005. When the stress increases from *σ*_tr_ = 98.4 MPa to *σ* = 180 MPa, the tangent modulus remains constant of *E*_p_ = 230.9 GPa with *η* = 0.005.

Under *σ*_s_ = 200 MPa, the initial strain is *ε*_0_ = 0.0174%; the initial tangent modulus is *E*_p_ = 229.6 GPa, the degradation rate of tangent modulus is 2.8% compared with original specimen and the fiber broken fraction is *P* = 0.048. With increasing stress to *σ*_tr_ = 118.8 MPa, the tangent modulus decreases to *E*_p_ = 215.4 GPa corresponding to *η* = 0.023. When the stress increases from *σ*_tr_ = 118.8 MPa to *σ* = 200 MPa, the tangent modulus remains constant of *E*_p_ = 217.8 GPa with *η* = 0.023.

Under *σ*_s_ = 220 MPa, the initial strain is *ε*_0_ = 0.033%; the initial tangent modulus is *E*_p_ = 220.1 GPa, the degradation rate of tangent modulus is 6.8% compared with original specimen and the fiber broken fraction is *P* = 0.082. With increasing stress to *σ*_tr_ = 139 MPa, the tangent modulus decreases to *E*_p_ = 184.5 GPa corresponding to *η* = 0.07. When the stress increases from *σ*_tr_ = 139 MPa to *σ* = 220 MPa, the tangent modulus remains constant of *E*_p_ = 189.2 GPa with *η* = 0.071.

**Figure 15:**
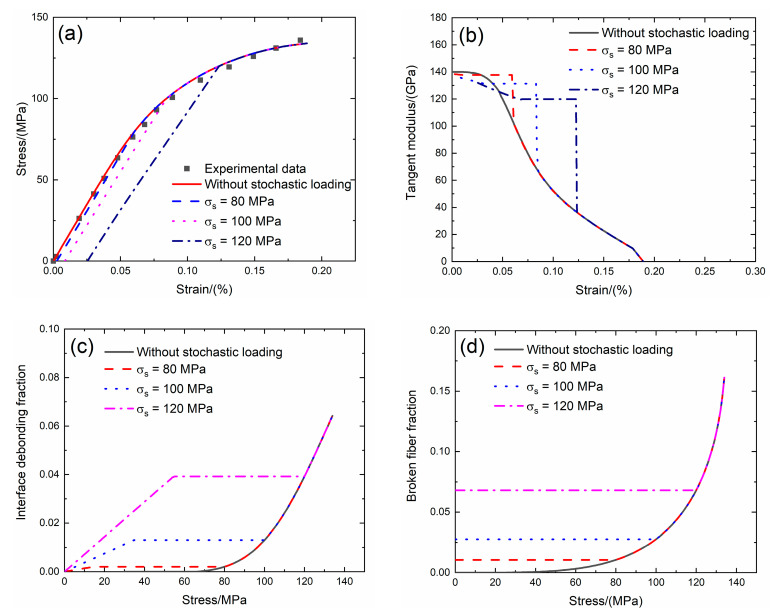
(**a**) The tensile stress–strain curves; (**b**) the tangent modulus versus strain curves; (**c**) the interface debonding fraction versus stress curves; and (**d**) the broken fibers fraction versus stress curves of 2D SiC/SiC composite for conditions without stochastic loading and with stochastic loading of *σ*_s_ = 80, 100 and 120 MPa.

For 2D SiC/SiC composite, the tensile stress–strain curves, tangent modulus versus strain curves, interface debonding fraction and broken fiber fraction versus stress curves without stochastic loading and with stochastic loading at *σ*_s_ = 80, 100 and 120 MPa at room temperature are shown in Figure 15 and Table 8.

Under *σ*_s_ = 80 MPa, the initial strain is *ε*_0_ = 0.002%; the initial tangent modulus is *E*_p_ = 138.3 GPa, the degradation rate of tangent modulus is 1.2% compared with original specimen and the fiber broken fraction is *P* = 0.01. With increasing stress to *σ*_tr_ = 13.2 MPa, the tangent modulus decreases to *E*_p_ = 137.6 GPa corresponding to *η* = 0.001. When stress increases from *σ*_tr_ = 13.2 MPa to *σ* = 80 MPa, the tangent modulus remains constant of *E*_p_ = 137.7 GPa with *η* = 0.002.

Under *σ*_s_ = 100 MPa, the initial strain is *ε*_0_ = 0.008%; the initial tangent modulus is *E*_p_ = 135.6 GPa, the degradation rate of tangent modulus is 3.1% compared with original specimen and the fiber broken fraction is *P* = 0.027. With increasing stress to *σ*_tr_ = 32.4 MPa, the tangent modulus decreases to *E*_p_ = 131 GPa corresponding to *η* = 0.012. When stress increases from *σ*_tr_ = 32.4 MPa to *σ* = 100 MPa, the tangent modulus remains constant of *E*_p_ = 131.3 GPa with *η* = 0.013.

Under *σ*_s_ = 120 MPa, the initial strain is *ε*_0_ = 0.025%; the initial tangent modulus is *E*_p_ = 131.9 GPa, the degradation rate of tangent modulus is 5.8% compared with original specimen and the fiber broken fraction is *P* = 0.068. With increasing stress to *σ*_tr_ = 52.8 MPa, the tangent modulus decreases to *E*_p_ = 119.5 GPa corresponding to *η* = 0.038. When stress increases from *σ*_tr_ = 52.8 MPa to *σ* = 120 MPa, the tangent modulus remains constant of *E*_p_ = 119.5 GPa with *η* = 0.039.

### 4.3. 2D SiC/SiC under Stochastic Loading of 80, 100 and 120 MPa

**Figure 16:**
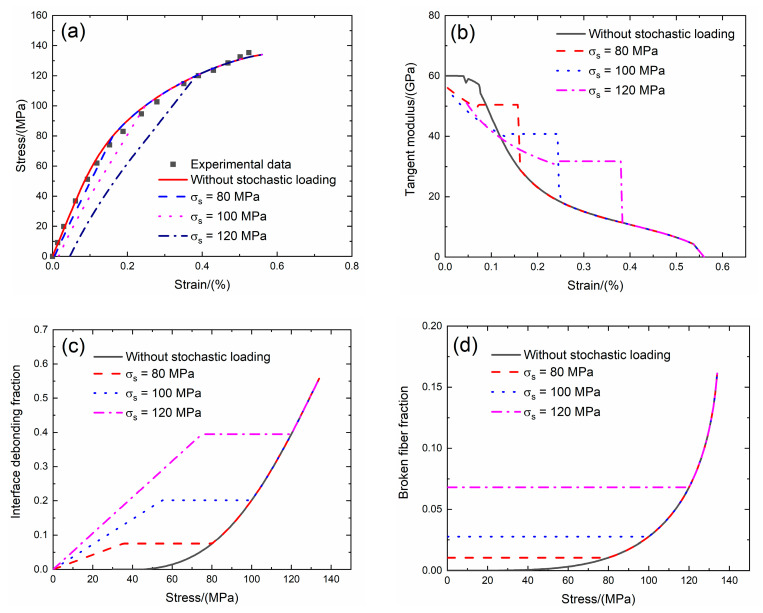
(**a**) The tensile stress–strain curves; (**b**) the tangent modulus versus strain curves; (**c**) the interface debonding fraction versus stress curves; and (**d**) the broken fibers fraction versus stress curves of 2D SiC/SiC composite for conditions without stochastic loading and with stochastic loading of *σ*_s_ = 80, 100 and 120 MPa.

Guo and Kagawa [6] investigated the tensile behavior of 2D plain-woven fabric SiC/SiC composite fabricated by the PIP process. The quasi-static tensile test was conducted under displacement control with the rate of 0.5 mm/min. The tensile stress–strain curves, tangent modulus versus strain curves, interface debonding fraction and broken fiber fraction versus stress curves without stochastic loading and with stochastic loading at *σ*_s_ = 80, 100 and 120 MPa at room temperature are shown in Figure 16 and Table 9.

Under *σ*_s_ = 80 MPa, the initial strain is *ε*_0_ = 0.005%; the initial tangent modulus is *E*_p_ = 56 GPa, the degradation rate of tangent modulus is 6.7% compared with original specimen and the fiber broken fraction is *P* = 0.01. With increasing stress to *σ*_tr_ = 33.6 MPa, the tangent modulus decreases to *E*_p_ = 49.4 GPa corresponding to *η* = 0.07. When stress increases from *σ*_tr_ = 33.6 MPa to *σ* = 80 MPa, the tangent modulus remains constant of *E*_p_ = 50.4 GPa with *η* = 0.074.

Under *σ*_s_ = 100 MPa, the initial strain is *ε*_0_ = 0.016%; the initial tangent modulus is *E*_p_ = 53.5 GPa, the degradation rate of tangent modulus is 11% compared with original specimen and the fiber broken fraction is *P* = 0.027. With increasing stress to *σ*_tr_ = 52.8 MPa, the tangent modulus decreases to *E*_p_ = 39.7 GPa corresponding to *η* = 0.19. When stress increases from *σ*_tr_ = 52.8 MPa to *σ* = 100 MPa, the tangent modulus remains constant of *E*_p_ = 40.7 GPa with *η* = 0.2.

Under *σ*_s_ = 120 MPa, the initial strain is *ε*_0_ = 0.046%; the initial tangent modulus is *E*_p_ = 51 GPa, the degradation rate of tangent modulus is 15% compared with original specimen and the fiber broken fraction is *P* = 0.068. With increasing stress to *σ*_tr_ = 73.2 MPa, the tangent modulus decreases to *E*_p_ = 30.8 GPa corresponding to *η* = 0.39. When stress increases from *σ*_tr_ = 73.2 MPa to *σ* = 120 MPa, the tangent modulus remains constant of *E*_p_ = 31.7 GPa with *η* = 0.395.

### 4.4. 2D SiC/SiC under Stochastic Loading of 180, 220, 260 and 300 MPa

**Figure 17:**
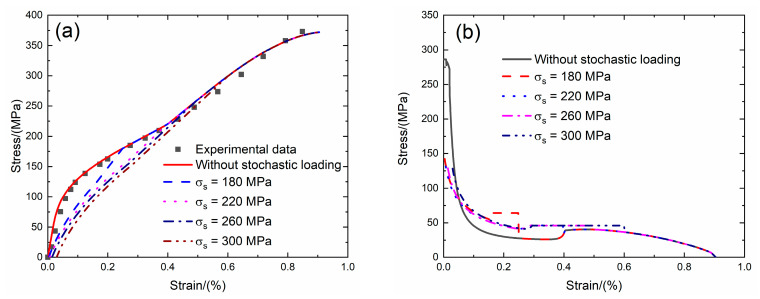

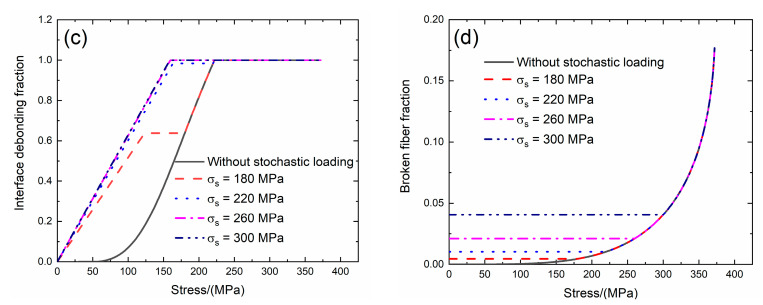
(**a**) The tensile stress–strain curves; (**b**) the tangent modulus versus strain curves; (**c**) the interface debonding fraction versus stress curves; and (**d**) the broken fibers fraction versus stress curves of 2D SiC/SiC composite for conditions without stochastic loading and with stochastic loading of *σ*_s_ = 180, 220, 260 and 300 MPa.

Morscher [7] investigated the tensile behavior of 2D SiC/SiC composite at room temperature. The tensile test was conducted under load control. The tensile stress–strain curves, tangent modulus versus strain curves, interface debonding fraction and broken fiber fraction versus stress curves without stochastic loading and with stochastic loading at *σ*_s_ = 180, 220, 260 and 300 MPa are shown in Figure 17 and Table 10.

Under *σ*_s_ = 180 MPa, the initial strain is *ε*_0_ = 0.003%; the initial tangent modulus is *E*_p_ = 142.3 GPa, the degradation rate of tangent modulus is 50% compared with original specimen and the fiber broken fraction is *P* = 0.004. With increasing stress to *σ*_tr_ = 122 MPa, the tangent modulus decreases to *E*_p_ = 53.9 GPa corresponding to *η* = 0.63. When stress increases from *σ*_tr_ = 122 MPa to *σ* = 180 MPa, the tangent modulus remains constant of *E*_p_ = 63.9 GPa with *η* = 0.638.

Under *σ*_s_ = 220 MPa, the initial strain is *ε*_0_ = 0.007%; the initial tangent modulus is *E*_p_ = 131.4 GPa, the degradation rate of tangent modulus is 54% compared with original specimen and the fiber broken fraction is *P* = 0.01. With increasing stress to *σ*_tr_ = 162 MPa, the tangent modulus decreases to *E*_p_ = 42 GPa corresponding to *η* = 0.63. When stress increases from *σ*_tr_ = 122 MPa to *σ* = 180 MPa, the tangent modulus remains constant of *E*_p_ = 63.9 GPa with *η* = 0.97.

Under *σ*_s_ = 260 MPa, the initial strain is *ε*_0_ = 0.014%; the initial tangent modulus is *E*_p_ = 128.7 GPa, the degradation rate of tangent modulus is 55% compared with original specimen and the fiber broken fraction is *P* = 0.021. With increasing stress to *σ*_tr_ = 150 MPa, the tangent modulus decreases to *E*_p_ = 41.3 GPa corresponding to *η* = 0.93. When stress increases from *σ*_tr_ = 150 MPa to *σ* = 260 MPa, the tangent modulus remains constant of *E*_p_ = 46 GPa with *η* = 1.0.

Under *σ*_s_ = 300 MPa, the initial strain is *ε*_0_ = 0.029%; the initial tangent modulus is *E*_p_ = 128.3 GPa, the degradation rate of tangent modulus is 55.1% compared with original specimen and the fiber broken fraction is *P* = 0.04. With increasing stress to *σ*_tr_ = 148 MPa, the tangent modulus decreases to *E*_p_ = 41.3 GPa corresponding to *η* = 0.93. When stress increases from *σ*_tr_ = 148 MPa to *σ* = 300 MPa, the tangent modulus remains constant of *E*_p_ = 46 GPa with *η* = 1.0.

## 5. Conclusions

In this paper, the effect of stochastic loading on tensile damage and fracture of fiber-reinforced CMCs is investigated. A micromechanical constitutive model is developed considering multiple damage mechanisms under tensile loading. The relationship between stochastic stress, tangent modulus, interface debonding and fiber broken is established. The effects of fiber volume, interface shear stress, interface debonding energy, saturation matrix crack spacing and fiber strength on tensile stress–strain curve, tangent modulus, interface debonding fraction and fiber broken fraction are analyzed. The experimental tensile damage and fracture of unidirectional and 2D SiC/SiC composites subjected to different stochastic loading stress are predicted.

(1) When fiber volume increases, the initial composite strain decreases, the initial tangent modulus increases, the transition stress for interface debonding decreases and the initial fiber broken fraction decreases;
(2) When the interface shear stress increases, the initial composite strain remains the same, the initial tangent modulus increases, the transition stress for interface debonding remains the same and the initial fiber broken fraction remains the same;
(3) When the interface debonding energy increases, the initial composite strain, tangent modulus and broken fiber fraction remain the same and the transition stress for interface debonding decreases;
(4) When saturation matrix crack spacing increases, the initial composite strain decreases, the initial tangent modulus increases and the transition stress for interface debonding and initial fiber broken fraction remain the same;
(5) When the fiber strength increases, the initial composite strain and fiber broken fraction decrease and the initial tangent composite modulus and transition stress for interface debonding remains the same.

## Figures and Tables

**Table 1:** The effect of the fiber volume (*V*_f_ = 0.3 and 0.35) on tensile stress–strain curve, tangent modulus, interface debonding fraction and broken fiber fraction of SiC/SiC composite subjected to stochastic loading of *σ*_s_ = 200, 300 and 350 MPa.

*V*_f_ = 0.3	*σ*_s_ = 200 MPa			
	*ε*_0_/(%)	*E*_p_/(GPa)	*σ*_tr_/(MPa)	*P*/(%)
	0.00161	268	79.2	0.005
	*σ*_s_ = 300 MPa			
	*ε*_0_/(%)	*E*_p_/(GPa)	*σ*_tr_/(MPa)	*P*/(%)
	0.0098	229	178.2	0.028
	*σ*_s_ = 350 MPa			
	*ε*_0_/(%)	*E*_p_/(GPa)	*σ*_tr_/(MPa)	*P*/(%)
	0.023	228	231	0.060
*V*_f_ = 0.35	*σ*_s_ = 200 MPa			
	*ε*_0_/(%)	*E*_p_/(GPa)	*σ*_tr_/(MPa)	*P*/(%)
	0.0009	281	55	0.0027
	*σ*_s_ = 300 MPa			
	*ε*_0_/(%)	*E*_p_/(GPa)	*σ*_tr_/(MPa)	*P*/(%)
	0.0047	249	156.2	0.014
	*σ*_s_ = 350 MPa			
	*ε*_0_/(%)	*E*_p_/(GPa)	*σ*_tr_/(MPa)	*P*/(%)
	0.0095	248	206.8	0.028

**Table 2:** The effect of interface shear stress (*τ*_i_ = 15 and 20 MPa) on tensile stress–strain curve, tangent modulus, interface debonding fraction and broken fiber fraction of SiC/SiC composite subjected to stochastic loading of *σ*_s_ = 200, 230 and 250 MPa.

*τ*_i_ = 15 MPa	*σ*_s_ = 200 MPa			
	*ε*_0_/(%)	*E*_p_/(GPa)	*σ*_tr_/(MPa)	*P*/(%)
	0.00943	195.6	125.4	0.028
	*σ*_s_ = 230 MPa			
	*ε*_0_/(%)	*E*_p_/(GPa)	*σ*_tr_/(MPa)	*P*/(%)
	0.021	163	154	0.056
	*σ*_s_ = 250 MPa			
	*ε*_0_/(%)	*E*_p_/(GPa)	*σ*_tr_/(MPa)	*P*/(%)
	0.038	149.5	173.8	0.092
*τ*_i_ = 20 MPa	*σ*_s_ = 200 MPa			
	*ε*_0_/(%)	*E*_p_/(GPa)	*σ*_tr_/(MPa)	*P*/(%)
	0.00943	215	125.4	0.028
	*σ*_s_ = 230 MPa			
	*ε*_0_/(%)	*E*_p_/(GPa)	*σ*_tr_/(MPa)	*P*/(%)
	0.021	185	154	0.056
	*σ*_s_ = 250 MPa			
	*ε*_0_/(%)	*E*_p_/(GPa)	*σ*_tr_/(MPa)	*P*/(%)
	0.038	171.7	173.8	0.092

**Table 3:** The effect of interface debonding energy (ζ_d_ = 0.1 and 0.3 J/m^2^) on tensile stress–strain curve, tangent modulus, interface debonding fraction and broken fiber fraction of SiC/SiC composite subjected to stochastic loading of *σ*_s_ = 180, 220 and 250 MPa.

*ζ*_d_ = 0.1 J/m^2^	*σ*_s_ = 180 MPa			
	*ε*_0_/(%)	*E*_p_/(GPa)	*σ*_tr_/(MPa)	*P*/(%)
	0.00537	251.5	140.8	0.018
	*σ*_s_ = 220 MPa			
	*ε*_0_/(%)	*E*_p_/(GPa)	*σ*_tr_/(MPa)	*P*/(%)
	0.0161	209	182.6	0.045
	*σ*_s_ = 250 MPa			
	*ε*_0_/(%)	*E*_p_/(GPa)	*σ*_tr_/(MPa)	*P*/(%)
	0.038	188.5	211.2	0.092
*ζ*_d_ = 0.3 J/m^2^	*σ*_s_ = 180 MPa			
	*ε*_0_/(%)	*E*_p_/(GPa)	*σ*_tr_/(MPa)	*P*/(%)
	0.00537	251.5	118.8	0.018
	*σ*_s_ = 220 MPa			
	*ε*_0_/(%)	*E*_p_/(GPa)	*σ*_tr_/(MPa)	*P*/(%)
	0.0161	209	158.4	0.045
	*σ*_s_ = 250 MPa			
	*ε*_0_/(%)	*E*_p_/(GPa)	*σ*_tr_/(MPa)	*P*/(%)
	0.038	188.5	189.2	0.092

**Table 4:** The effect of the saturation matrix crack spacing (*l*_s_ = 200 and 250 μm) on tensile stress–strain curve, tangent modulus, interface debonding fraction and broken fiber fraction of SiC/SiC composite subjected to stochastic loading of *σ*_s_ = 180, 220 and 250 MPa.

*l*_s_ = 200 μm	*σ*_s_ = 180 MPa			
	*ε*_0_/(%)	*E*_p_/(GPa)	*σ*_tr_/(MPa)	*P*/(%)
	0.00567	229.8	103.4	0.018
	*σ*_s_ = 220 MPa			
	*ε*_0_/(%)	*E*_p_/(GPa)	*σ*_tr_/(MPa)	*P*/(%)
	0.0168	180.3	143	0.045
	*σ*_s_ = 250 MPa			
	*ε*_0_/(%)	*E*_p_/(GPa)	*σ*_tr_/(MPa)	*P*/(%)
	0.038	157.7	173.8	0.092
*l*_s_ = 250 μm	*σ*_s_ = 180 MPa			
	*ε*_0_/(%)	*E*_p_/(GPa)	*σ*_tr_/(MPa)	*P*/(%)
	0.00549	242.3	103.4	0.018
	*σ*_s_ = 220 MPa			
	*ε*_0_/(%)	*E*_p_/(GPa)	*σ*_tr_/(MPa)	*P*/(%)
	0.0164	196.8	143	0.045
	*σ*_s_ = 250 MPa			
	*ε*_0_/(%)	*E*_p_/(GPa)	*σ*_tr_/(MPa)	*P*/(%)
	0.038	174.8	173.8	0.092

**Table 5:** The effect of the fiber strength (*σ*_c_ = 2.0 and 2.5 GPa) on tensile stress–strain curve, tangent modulus, interface debonding fraction and broken fiber fraction of SiC/SiC composite subjected to stochastic loading of *σ*_s_ = 180, 220 and 250 MPa.

*σ*_c_ = 2.0 GPa	*σ*_s_ = 180 MPa			
	*ε*_0_/(%)	*E*_p_/(GPa)	*σ*_tr_/(MPa)	*P*/(%)
	0.0065	251.5	103.4	0.022
	*σ*_s_ = 220 MPa			
	*ε*_0_/(%)	*E*_p_/(GPa)	*σ*_tr_/(MPa)	*P*/(%)
	0.02	209.6	143	0.058
	*σ*_s_ = 250 MPa			
	*ε*_0_/(%)	*E*_p_/(GPa)	*σ*_tr_/(MPa)	*P*/(%)
	0.059	188.5	173.8	0.138
*σ*_c_ = 2.5 GPa	*σ*_s_ = 180 MPa			
	*ε*_0_/(%)	*E*_p_/(GPa)	*σ*_tr_/(MPa)	*P*/(%)
	0.028	251.5	103.4	0.0087
	*σ*_s_ = 220 MPa			
	*ε*_0_/(%)	*E*_p_/(GPa)	*σ*_tr_/(MPa)	*P*/(%)
	0.007	209.6	143	0.02
	*σ*_s_ = 250 MPa			
	*ε*_0_/(%)	*E*_p_/(GPa)	*σ*_tr_/(MPa)	*P*/(%)
	0.015	188.5	173.8	0.036

**Table 6:** The tensile stress–strain curve, tangent modulus, interface debonding fraction and broken fiber fraction of 2D SiC/SiC composite subjected to stochastic loading of *σ*_s_ = 140, 180, 200 and 240 MPa.

*σ*_s_ = 140 MPa.			
*ε*_0_/(%)	*E*_p_/(GPa)	*σ*_tr_/(MPa)	*P*
0.001	288	63.8	0.006
*σ*_s_ = 180 MPa			
*ε*_0_/(%)	*E*_p_/(GPa)	*σ*_tr_/(MPa)	*P*
0.005	251	103.4	0.018
*σ*_s_ = 180 MPa			
*ε*_0_/(%)	*E*_p_/(GPa)	*σ*_tr_/(MPa)	*P*
0.009	229.5	123.2	0.028
*σ*_s_ = 180 MPa			
*ε*_0_/(%)	*E*_p_/(GPa)	*σ*_tr_/(MPa)	*P*
0.028	194.2	162.8	0.078

**Table 7:** The tensile stress–strain curve, tangent modulus, interface debonding fraction and broken fiber fraction of unidirectional SiC/SiC composite subjected to stochastic loading of *σ*_s_ = 140, 180, 200 and 220 MPa.

*σ*_s_ = 140 MPa			
*ε*_0_/(%)	*E*_p_/(GPa)	*σ*_tr_/(MPa)	*P*
0.002	235.9	58.8	0.01
*σ*_s_ = 180 MPa			
*ε*_0_/(%)	*E*_p_/(GPa)	*σ*_tr_/(MPa)	*P*
0.009	234	98.4	0.03
*σ*_s_ = 200 MPa			
*ε*_0_/(%)	*E*_p_/(GPa)	*σ*_tr_/(MPa)	*P*
0.017	229.6	118.8	0.048
*σ*_s_ = 220 MPa			
*ε*_0_/(%)	*E*_p_/(GPa)	*σ*_tr_/(MPa)	*P*
0.033	220.1	139	0.082

**Table 8:** The tensile stress–strain curve, tangent modulus, interface debonding fraction and broken fiber fraction of 2D SiC/SiC composite subjected to stochastic loading of *σ*_s_ = 80, 100 and 120 MPa.

*σ*_s_ = 80 MPa			
*ε*_0_/(%)	*E*_p_/(GPa)	*σ*_tr_/(MPa)	*P*
0.002	138.3	13.2	0.01
*σ*_s_ = 100 MPa			
*ε*_0_/(%)	*E*_p_/(GPa)	*σ*_tr_/(MPa)	*P*
0.008	135.6	32.4	0.027
*σ*_s_ = 120 MPa			
*ε*_0_/(%)	*E*_p_/(GPa)	*σ*_tr_/(MPa)	*P*
0.025	131.9	52.8	0.068

**Table 9:** The tensile stress–strain curve, tangent modulus, interface debonding fraction and broken fiber fraction of 2D SiC/SiC composite subjected to stochastic loading of *σ*_s_ = 80, 100 and 120 MPa.

*σ*_s_ = 80 MPa			
*ε*_0_/(%)	*E*_p_/(GPa)	*σ*_tr_/(MPa)	*P*
0.005	56	33.6	0.01
*σ*_s_ = 100 MPa			
*ε*_0_/(%)	*E*_p_/(GPa)	*σ*_tr_/(MPa)	*P*
0.016	53.5	52.8	0.027
*σ*_s_ = 120 MPa			
*ε*_0_/(%)	*E*_p_/(GPa)	*σ*_tr_/(MPa)	*P*
0.046	51	73.2	0.068

**Table 10:** The tensile stress–strain curve, tangent modulus, interface debonding fraction and broken fiber fraction of 2D SiC/SiC composite subjected to stochastic loading of *σ*_s_ = 180, 220, 260 and 300 MPa.

*σ*_s_ = 180 MPa			
*ε*_0_/(%)	*E*_p_/(GPa)	*σ*_tr_/(MPa)	*P*
0.003	142.3	122	0.004
*σ*_s_ = 220 MPa			
*ε*_0_/(%)	*E*_p_/(GPa)	*σ*_tr_/(MPa)	*P*
0.007	131.4	162	0.01
*σ*_s_ = 260 MPa			
*ε*_0_/(%)	*E*_p_/(GPa)	*σ*_tr_/(MPa)	*P*
0.014	128.7	150	0.021
*σ*_s_ = 300 MPa			
*ε*_0_/(%)	*E*_p_/(GPa)	*σ*_tr_/(MPa)	*P*
0.029	128.3	148	0.04
